# An Analysis of 60 Years of Autopsy Data from Zhejiang University in Hangzhou, China

**DOI:** 10.1371/journal.pone.0112500

**Published:** 2014-11-26

**Authors:** Keqing Zhu, Haijia Feng, Yinhan Xu, Zhengrong Mao, Wei Zhang, Jian Chen, Liqing Ma, Minche Chen, Qiunian Shi, Suojiang Zhang

**Affiliations:** 1 Department of Pathology, Zhejiang University School of Medicine, Hangzhou, China; 2 Department of Neuroscience, Zhejiang University School of Medicine, Hangzhou, China; 3 China Brain Bank, Zhejiang University School of Medicine, Hangzhou, China; Zhejiang University, China

## Abstract

**Context:**

The autopsy rate gradually decreased during 1950–1999, and increased during the most recent decade (2000–2009). The diagnostic inaccuracy rate was continuously high during the 60 years.

**Objective:**

To investigate disagreement between the pathological and clinical diagnosis during 60 years (1950–2009).

**Data Sources:**

A 60-year retrospective study was carried out on the 4140 autopsy cases performed in Zhejiang University School of Medicine.

**Results:**

The highest number of cases was 1037 during 1960–1969, while the lowest was 102 during 1990–1999. During the 1999–2009 period, 978 cases were completed, which ranked second within the 60 years. The total clinical misdiagnosis rate was 46.38%, while the highest was 73.82% in 2000–2009. During the 60 years, the diseases associated with highest diagnostic inaccuracy rates were circulatory diseases (76.97%), cancer (60.99%), and brain diseases (54.48%). The invasive fungal infection rate was 1.84% of the 4140 cases, and the diagnostic inaccuracy rate for this condition reached as high as 86.10%. In the autopsied disease spectrum over the 60 years, the most common diseases were respiratory (1349, 32.58%), circulatory (495, 11.96%), and brain diseases (424, 10.24%).

**Conclusion:**

Although the number of autopsies decreased from 1950 to 1999, it increased from 2000 to 2009, while the discordance rate between clinical and autopsy diagnosis remained high throughout.

## Introduction

In China, hospitals are graded, and the Ministry of Health requires that hospitals belonging to the III and II grades must attain autopsy rates of at least 15% and 10%, respectively. In reality, these percentages are not achieved in many hospitals. For example, the number of autopsies conducted between 1990 and 1999 in Hangzhou (population 6 million), was only 102, which translates into an average of only 10 per year. In a span of 10 years, hospitals in Hangzhou have had only between 1 and 3 autopsies, or even less. The number of autopsies conducted is disproportionate to the size and grading of the hospitals in Hangzhou. In addition, many hospitals do not conduct clinicopathological conferences, resulting in problems in the training of new physicians as well as the development of clinical medicine. Here, an analysis of 4140 autopsies in the past 60 years conducted at Zhejiang University School of Medicine in Hangzhou between 1950 and 2009 was carried out to focus attention on the field.

## Methods

### Case information

Cases was obtained concerning 4140 autopsies conducted between 1950 and 2009 at Zhejiang University School of Medicine (known as Zhejiang Medical University prior to 1999). Disease analysis and the categorization of pathological diagnoses were in accord with the WHO guidelines on “International Standards for Diseases and Injury”. The autopsy center identifies the death reason, at the same time, it is the teaching and scientific research unit, and will carry out related photography, video, teaching and research activities before and after the inspection process. The patient family members understand and support the above works. The study was reviewed and approved by Zhejiang University School of Medicine Ethics Committee before the study began. The written informed consent was given by the next of kin/caregiver in the case of children or the deceased, for their clinical records to be used in this study. The patient records/information was anonymized and de-identified prior to analysis.

### Statistical data

The number of autopsy cases in the 60 years, the gender and age of the autopsied individuals, comparison of the autopsy and clinical diagnoses, and the distribution of autopsy-determined fatal diseases were determined. In the event of a list of possible clinical diagnoses, only the first three were taken into account for statistical purposes. In the event where any one item from clinical diagnosis overlapped with an item from the autopsy-determined diagnosis, the two diagnoses were said to concur, otherwise being taken as misdiagnosis. We summarized and compared our findings with autopsy reports from some of the other major medical centers in China.

### Age grouping

Fetuses of 20 weeks to neonates 28 days after birth comprised the perinatal group, >28 days to 7 years the infant group, >7 to 18 years the child and adolescent group, and >18 years the adult group. Further categorization was done according to the location of the fatal pathological change. At the same time, cases involving tuberculosis were categorized under infectious disease. Other cases that did not fall into the above categories formed the last group (others).

## Results

A total of 4140 autopsy cases were conducted between 1950 and 2009. Of these, 2785 were male and 1355 female (ratio 2.06∶1). The largest number of cases (1037) occurred between 1960 and 1969, giving an average of >100 per year. The fewest (102) were recorded between 1990 and 1999, giving an average of only 10 per year. The number of cases conducted at Zhejiang University School of Medicine reflected the standard number of autopsies in Hangzhou (Table 1).

**Table 1:** The ages and genders categorization of 4140 autopsied individuals in the different times.

Year	No.	Male	Female	Perinatal (%)	Infant (%)	Child and adolescent (%)	Adult (%)
1950–1959	873	582	291	174 (19.93)	334 (38.26)	64 (7.33)	301 (34.48)
1960–1969	1037	682	355	207 (19.96)	409 (39.44)	72 (6.94)	349 (33.65)
1970–1979	595	384	211	98 (16.47)	198 (33.28)	61 (10.25)	238 (40.00)
1980–1989	555	371	184	284 (51.17)	135 (24.32)	22 (3.96)	114 (20.54)
1990–1999	102	65	37	44 (43.14)	17 (16.67)	4 (3.92)	37 (36.27)
2000–2009	978	701	277	104 (10.63)	86 (8.79)	38 (3.89)	750 (76.69)
Total	4140	2785	1355	911 (22.00)	1179 (28.48)	261 (6.30)	1789 (43.21)

The overall misdiagnosis rate was 46.38%. According to the statistics, this rate was highest between 2000 and 2009, reaching 73.82%, and lowest between 1980 and 1989, falling to 30.63%. The misdiagnosis rate between 1990 and 1999 was 41.18%. During the 60 years, the highest misdiagnosis rate was for circulatory diseases (76.97%), followed by tumors (60.99%) and brain diseases (54.48%). This shows that even with the advances in medical technology and diagnostic methods, the rate of clinical misdiagnosis remained rather high (Table 2).

**Table 2:** Comparison of the autopsy-determined pathological diagnosis and the clinical diagnosis.

Disease	1950∼1959	1960∼1969	1970∼1979	1980∼1989	1990∼1999	2000∼2009	Total	No to total cases ratio(%)	Error rate (%)
Cancer	35(18)	51(30)	42(33)	29(18)	8(4)	17(8)	182(111)	4.40	60.99
Respiratory	346(70)	299(92)	134(48)	203(34)	39(9)	328(283)	1349(536)	32.58	39.73
Alimentary	71(35)	127(54)	82(33)	61(20)	9(3)	63(38)	413(183)	9.98	44.31
Circulatory	40(24)	96(65)	67(43)	36(15)	15(10)	241(224)	495(381)	11.96	76.97
Urogenital	25(12)	24(11)	11(5)	13(3)	2(0)	20(12)	95(43)	2.29	45.26
Nervous	66(30)	70(22)	50(25)	58(27)	7(6)	173(121)	424(231)	10.24	54.48
Blood	33(10)	55(14)	35(10)	12(3)	2(1)	8(5)	125(43)	3.02	34.40
Congenital	32(13)	44(17)	36(14)	68(21)	9(5)	1(1)	190(71)	4.59	37.37
Infectious	127(48)	157(32)	58(16)	21(10)	4(2)	2(1)	369(109)	8.91	29.54
Parasitic	29(14)	17(8)	13(6)	2(2)	—	2(1)	63(31)	1.52	49.21
Sepsis	30(13)	82(26)	43(11)	37(13)	3(1)	11(6)	206(70)	4.98	33.98
Others	39(10)	35(9)	24(13)	15(4)	4(1)	112(74)	229(111)	5.53	48.47
Total	873(297)	1037(380)	595(257)	555(170)	102(42)	978 (774)	4140(1920)	100.00	46.38
Error rate(%)	34.02	36.64	43.19	30.63	41.18	73.82	46.38		

According to the disease categorization, the tumor misdiagnosis rate was 60.99%. Among the 4140 cases, 182 involved malignant tumors. Of these, 71 were correctly diagnosed (39.01%), 81 were misdiagnosed (44.51%), and 30 were undiagnosed (16.48%). The five most common locations of undiagnosed or misdiagnosed cancers were the brain (95.00%), endocrine system (92.30%), urogenital system (75.00%), blood (56.36%) and respiratory tract (46.67%) (Table 3). The discordance rate between clinical and autopsy diagnosis is similar to other report.^2^

**Table 3:** List of the distribution of all 182 cases of cancer as well as examples of misdiagnosis and undiagnosed diseases.

Position	number	misdiagnosis	undiagnosis	Error rate
Respiratory	30	12	2	46.67%
Digestive	37	7	5	32.43%
CNS	20	14	5	95.00%
Urogenital	16	5	7	75.00%
Blood	55	24	7	56.36%
Endocrine	13	10	2	92.30%
Breast	1	---	1	100.00%
Others	10	9	1	100.00%
Total	182	81	30	60.99%

Among the 4140 autopsies, there were 76 cases of deep fungal infection and the total misdiagnosis rate was 86.10% (Table 4). The rate of occurrence increased from 1950 to 1999: 0.24% in 1950–9, 2.51% in 1960–9, 4.03% in 1970–9, 3.06% in 1980–9, and 2.94% in 1990–9. From 2000 to 2009, there were only 4 fungal infection cases, the occurrence rate being 0.4%.

**Table 4:** List of all 76 invasive fungal infection cases during the 60 years autopsy cases.

Time	1950–1959	1960–1969	1970–1979	1980–1989	1990–1999	2000–2009	Total
Autopsy cases	873	1037	595	555	102	978	4140
Fungal infection	2	26	24	17	3	4	76
*ratio*	0.24%	2.51%	4.03%	3.06%	2.94%	0.41%	1.84%

## Discussion

**Figure 1:**
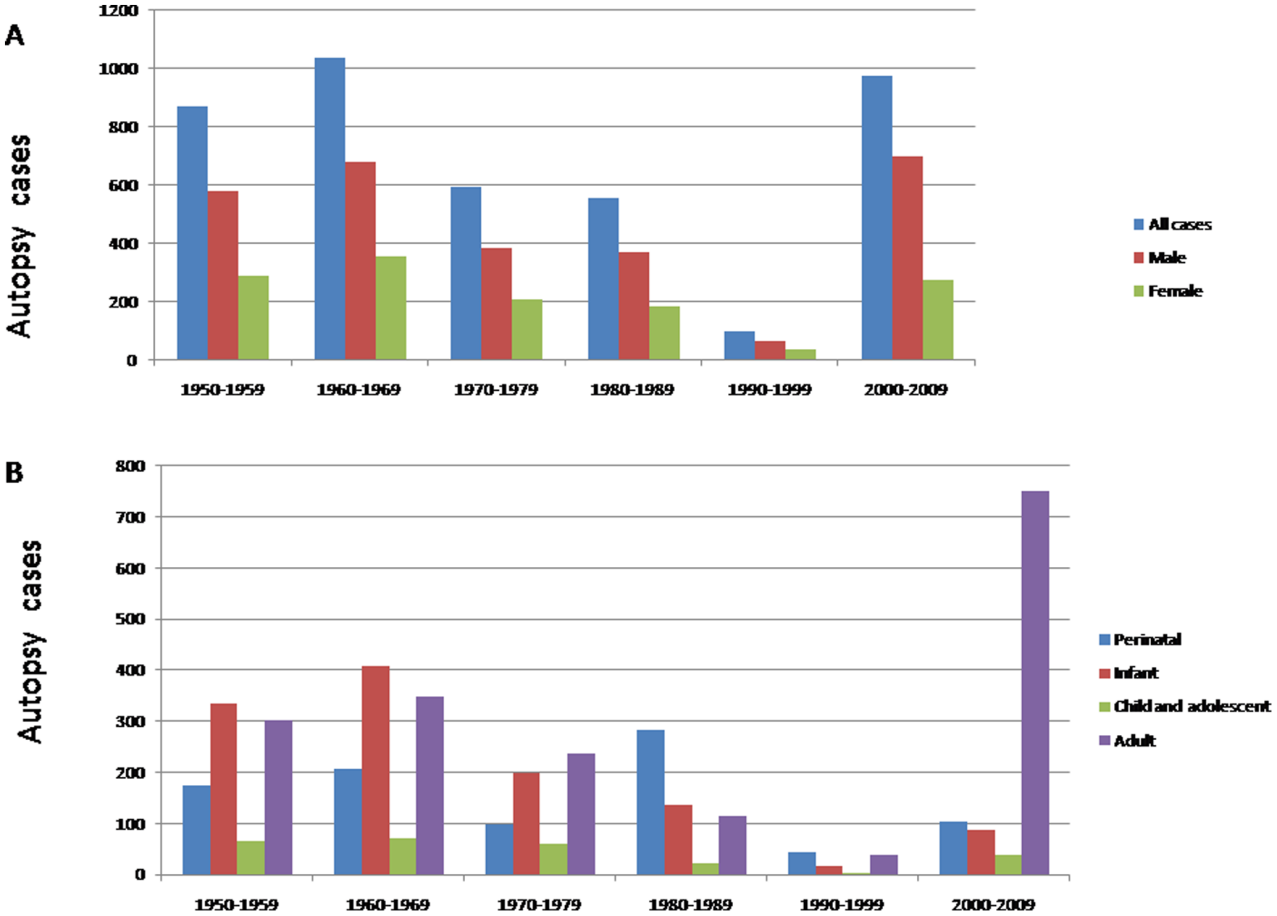
A total of 4140 autopsy cases were conducted between 1950 and 2009. Of these, 2785 were male and 1355 female. The largest number of cases occurred between 1960 and 1969. The fewest were recorded between 1990 and 1999(Figure 1A). The Adult and the infant groups are the major part in all cases (Figure 1B).

From 1950 to 1999, the autopsy rate declined steadily with a sharp decrease between 1990 and 1999 (Figure 1A–B), with an average of 10 cases per year, and the fewest being 5 cases in one year. The reasons for this decline are likely to be: (1) The social and hospital environment. The first refers to societal and traditional beliefs and habits, and the second to the fear of lawsuits brought against the hospital by the family of the deceased in the event of a clinical misdiagnosis confirmed at autopsy [1]. (2) Clinicians may avoid using advanced medical technology to confirm the clinical diagnosis. (3) Lack of emphasis by the hospital and lack of initiative by clinicians. The fact is that the 10–15% autopsy rate required by the Ministry of Health only exists on paper and not in reality. Hospitals have not encouraged the development of autopsy work. (4) The fee for conducting an autopsy and the subsequent arrangements must be borne by the family of the deceased, though the resources for the autopsy are provided by the hospital.

**Figure 2:**
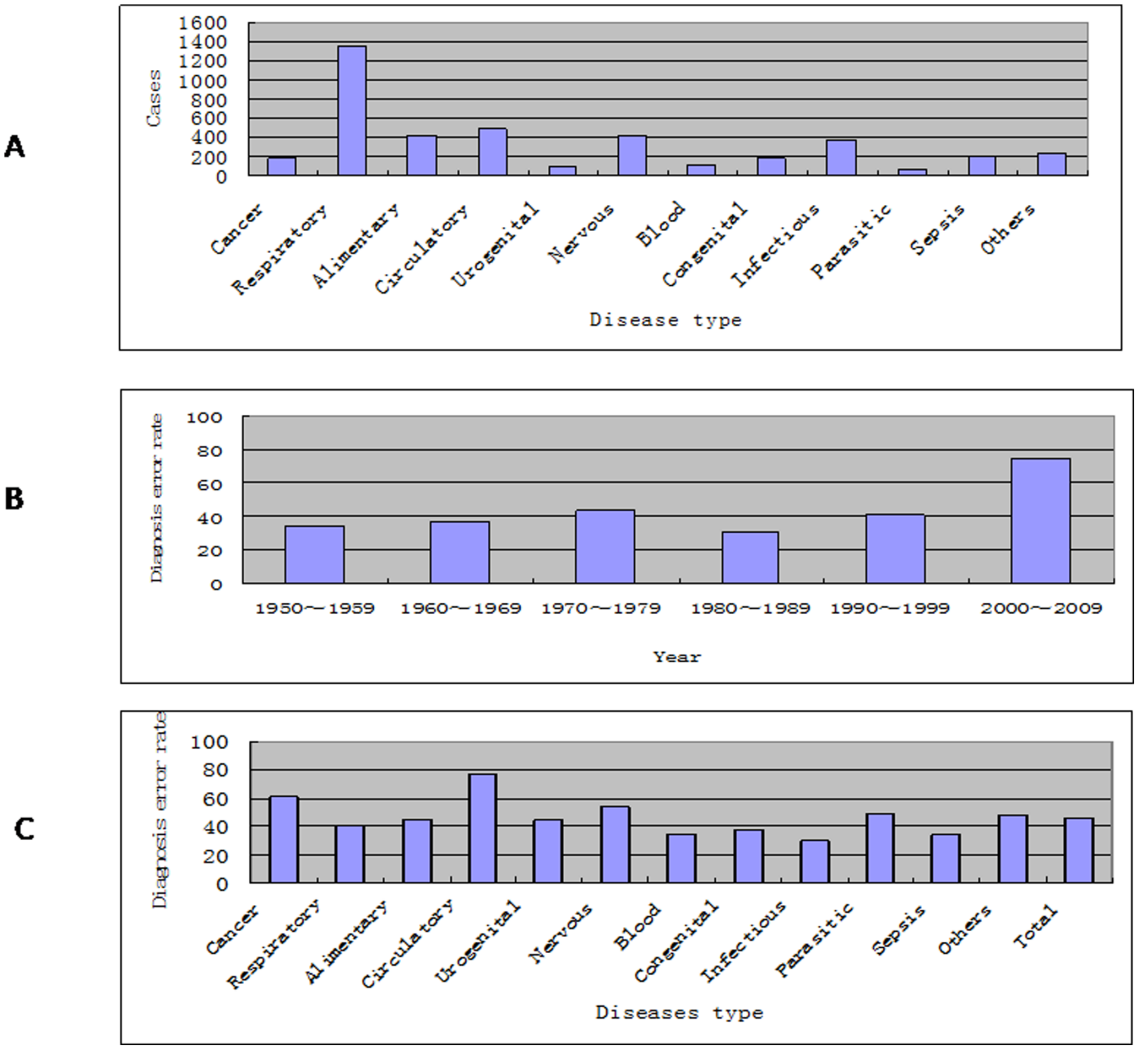
Within the 60 years, based on the disease distribution, respiratory diseases ranked the highest, followed by circulatory diseases and brain diseases (Figure 2A). In terms of the diagnostic error rate, the 2000–9 group was the highest. Second was the 1970–9 group, and third was the 1990–9 group (Figure 2B). In terms of the diseases, the most misdiagnosed were in the circulatory system. Ranking second among the categories with misdiagnoses was tumor, followed in descending order by parasitic diseases, and diseases of the genitourinary system, alimentary tract, and respiratory tract (Figure 2C).

Within the 60 years, based on the disease distribution, respiratory diseases ranked the highest (1349, 32.58%), followed by circulatory diseases (495, 11.96%) and brain diseases (424, 10.24%), and then diseases of the alimentary tract (413, 9.98%) and infections (369, 8.91%). These five categories accounted for 73.67% of the total cases (Figure 2A).

In terms of the diagnostic error rate, the 2000–9 group was the highest (73.82%). Second was the 1970–9 group at 43.19%, and third was the 1990–9 group at 41.18%. Even for the lowest group, the error rate was as high as 30.63%, in 1980–9 (Figure 2B). In terms of the diseases, the most misdiagnosed were in the circulatory system (error rate 76.97%). These mainly occurred in patients whose cause of death was unknown at the time of autopsy. The rate of tumor misdiagnosis was 60.99% (Figure 2C), higher than the national average (26.29%) and that overseas (44.00%) [2]. In the 182 cancer cases, the misdiagnosis rate ranked highest for brain cancers at 95.00% (19/20), followed by endocrine cancers at 92.30% (12/13), urogenital cancers at 75.00% (12/16), and blood cancers at 56.36% (31/55). Breast cancer (only 1 case) and the other cancers group (10/10) were not included (Table 3).

**Figure 3:**
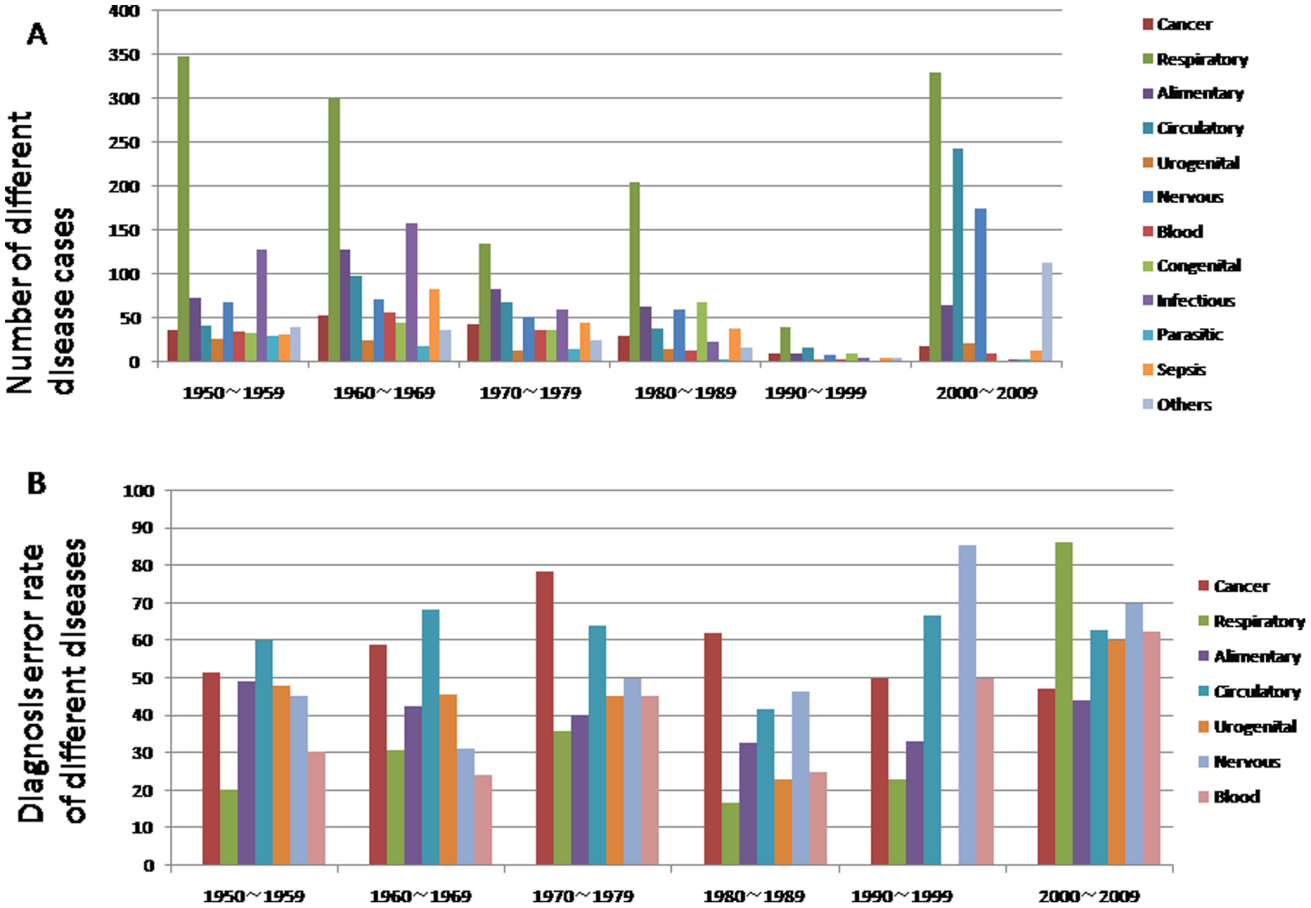
The number of different diseases in the different periods (Figure 3A). The different misdiagnosis rate for the different diseases between 1950 and 2009(Figure 3B).

Ranking third among the categories with misdiagnoses was brain diseases (231/424, 54.48%), followed in descending order by parasitic diseases (31/63, 60.99%), and diseases of the genitourinary system (43/95, 45.26%), alimentary tract (183/413, 44.31%), and respiratory tract (536/1349, 39.37%) (Figure 2C). The parasitic diseases mostly occurred between 1950 and 1959 (29 cases). There were only 2 cases each in 1980–9 and 2000–9, and none in 1990–9. This reflects improvements in hygiene and a decline in occurrence in Hangzhou (Figure 3A).

An improvement of the situation has occurred in recent years. In the most recent decade (2000–9), a total of 978 autopsies were conducted, nine-fold more than the 102 between 1990 and 1999. We found that cases of adult sudden death increased within the most recent decade. There were 72 in 2000–9, while there were only 118 in total from 1950 to 1999. Since most of the autopsies resulted from dissension between the relatives and the medical staff along with an unclear diagnosis before death, the misdiagnosis rate had evidently increased that decade. The misdiagnosis rate of 73.82% in 2000–9, the highest of all the decades, with 92.95% for diseases of the circulatory system (224/241), 86.28% for the respiratory tract (283/328), and 69.94% for the brain (121/173). The misdiagnosis rate was the highest for diseases of all three systems (Figure 3B).

In other major medical centers in China, the misdiagnosis rate was also high [3]–[5]. For example, the lowest rate was 20.00% reported in Guangzhou from 1958 to 1992. The findings from our analysis are similar to those reported by other groups (Table 5). The diagnosis technology has developed rapidly in the last sixty years, while the discordance rate has remained high in our cases. One reason is that many patients died because of sudden death with a short duration of hospitalization. In these cases, clinicians had to give the immediate and the underlying causes of death for patients dying under their care, sometimes lack of careful medical examination. That is one of the reasons that the discordance rate is particularly high for certain diseases, such as cardiovascular diseases. Adding CT/MRI/PETCT imaging results may help to reduce the misdiagnosis rate. The second reason is many cases came from grade I or grade II hospitals, whose medical levels are not as good as that of grade III hospitals. The misdiagnosis rate in grade I or grade II hospitals was higher than that of grade III hospitals. Thirdly, many cases consisted the diagnosis disputes between the relatives and the clinicians. At present, there exist increasing trend for medical malpractice disputes. In the case of medical disputes in the death, as the best evaluation and the objective and fair solution to the problem, the autopsy is necessary for doctors and families.

**Table 5:** Major diagnostic error rates in the different identical centers in China.

Year	Location	Autopsy	Error rate
1946–1980	Beijing	4738	20.74%
1958–1992	Guangzhou	4074	20.00%
1960–1995	Guangxi	1392	30.00%
1952–1987	Chengdu	6668	31.10%
1955–1999	Shanghai	2690	26.49%
1950–2009	Hangzhou	4140	46.38%

In US, the results of 53 distinct autopsy series over a 40-year period (from 1966 to 2002) show statistically significant decreases over time for major diagnostic errors detected at autopsy [6]. In China Hangzhou, during the 60 years from 1950 to 2009, the discordance rate between clinical and autopsy diagnosis remained high. Although the autopsy rate decreased from 1950 to 1999, both the autopsy number and the diagnostic error rate increased during the most recent decade (2000–2009). With sufficient emphasis and initiative by the patients' relatives and doctors, the field of autopsy can be improved. This will be beneficial both for patients and their families [7], and the advancement of practice and research in clinical medicine in China.
